# AI-Driven Analysis Unveils Functional Dynamics of Müller Cells in Retinal Autoimmune Inflammation

**DOI:** 10.1101/2025.02.28.640907

**Published:** 2025-03-10

**Authors:** Guangpu Shi, Vijayaraj Nagarajan, Rachel R. Caspi

**Affiliations:** Laboratory of Immunology, National Eye Institute, NIH, Bethesda 20892, USA

**Keywords:** Rodent, T Cells, SCassist, Autoimmunity, Cytokines, Chemokines

## Abstract

Müller cell is the most common type of glial cell in the human and mouse retina, playing a crucial role in maintaining retinal homeostasis. In addition to providing structural support to the retina, Müller cells can also supply trophic substances to retinal neurons, remove metabolic waste, mitigate oxidative stress, and promote synaptic activities. However, many roles of Müller cells remain largely unknown, particularly for those in the inflamed retina. In this article, we reanalyzed a single cell RNA-seq (scRNA-seq) dataset from Aire−/− mice, which exhibits autoimmune retinal inflammation, specifically focusing on Müller cells and T cells, identifying nine distinct Müller cell subgroups along with five T cell subgroups. Among them, three subgroups of Müller cells are activated Müller cells, representing over 60% Müller cells in the inflamed retina. Using SCassist - an Artificial Intelligence (AI) based workflow assistant for single-cell analysis, we constructed a comparison matrix to quantify the involvement of pathways characterizing the functions of each Müller cell subpopulation. The activated Müller cells primarily present a macrophage-like phenotype with or without augmentation of the known Müller cell functions. Trajectory analysis further identified two paths, validating the presence of these two phenotypes, governed by Neurod1 and Irf family transcription factors (TFs). We further inferred the interactions between Müller cells and T cells and observed that activated Müller cells do not exhibit extra chemoattraction to Th1 cells compared to other Müller cells but display nearly exclusive expression of immune checkpoint molecules, primarily targeting Th1 cells. Our findings open new avenues for understanding the specialized mechanisms of retinal pathogenic autoimmunity and identifying candidates to explore potential inhibitory pathways in the inflamed retina.

## Introduction

Müller cells, first described by Heinrich Müller, are the most common type of glial cells in the human and mouse retinas, followed by astroglia and microglia. Müller cells are the only glial cell type that, like retinal neurons, derives from retinal progenitor cells, whereas astrocytes and microglia migrate into the developing retina from external sources (1–3). Müller cells play a crucial role in maintaining retinal homeostasis. Currently, many roles of Müller cells in regulating retinal function remain largely unknown (4). What we have learned are that, in addition to providing structural support to the retina, ensuring the viability and stability of retinal cells, Müller cells supply trophic substances to neurons, provide antioxidative molecules to the retina, remove metabolic waste through phagocytosis, and sustain synaptic activity through neurotransmitter recycling (5–7). Ions and signaling molecules, including cAMP, are actively involved in these processes (4, 8).

Retinal inflammation is a central component of human uveitis, a pathogenic autoimmune condition and a major cause of blindness, contributing to 10–15% of severe visual impairment, particularly in the working age (9, 10). The term “uveitis” is used to define a group of eye disorders with intraocular inflammation, including diseases, such as sympathetic ophthalmia, birdshot chorioretinopathy, sarcoidosis, Behcet’s disease, and Vogt-Koyanagi-Harada disease (11–13). By utilizing animal models, we have gained valuable understanding of the pathophysiology of this condition; however, it remains unclear how retinal Müller cells respond to or modulate the inflammatory process in the affected retina (14–18). So far, much of the available information on Müller cell - T cell interaction has come from the in vitro studies, which have provided clear evidence that Müller cells can inhibit T cell activation, as demonstrated by the reduced IL-2 receptor expression on T cells and inhibited antigen-induced T cell proliferation following contact with Müller cells in co-culture systems (19–22). However, the role of Müller cells in retinal inflammation has not been fully explored in vivo.

Recent studies have demonstrated that Müller cells may interact with T cells by releasing chemokines or expressing cell surface molecules including immune checkpoint molecules, targeting their receptors on T cells (17, 18). Yet, these data were collected at the bulk Müller cell level, lacking subgroup-specific information, which might have overlooked critical details underlying Müller cell activities. Here we leveraged the availability of a scRNA-seq dataset from the inflamed retina of an Aire deficiency (Aire−/−) mouse model to explore the roles of Müller cell subgroups and their interactions with infiltrated T cells. Aire is expressed in medullary thymic epithelial cells, where it is responsible for maintaining central tolerance. Aire−/− mouse develops spontaneous and progressive uveoretinitis as part of a multiorgan autoimmune phenotype due to the loss of central tolerance to autoantigens (23, 24).

Subgroups of Müller cells in the inflamed retina remain unexplored, and no established nomenclature exists to facilitate our understanding of their functions. In this article, we used SCassist to construct a comparison matrix quantifying the involvement of pathways in each of nine Müller cell subgroups from Aire−/− retina to characterize their features. Five classic T cell subgroups were also identified alongside these Müller cells. SCassist (https://github.com/NIH-NEI/SCassist), an AI-based workflow assistant developed in our lab applies the power of the large language models (LLMs) like Google’s Gemini and Meta’s Llama3, allowing us to incorporate AI driven insights into the scRNA-seq analysis. With assistance from SCassist, we identified macrophage-like phenotype for the activated Müller cell subgroups, with or without augmented Müller cell functions. Further analysis discovered that Neurod1 and Irf family TFs are the crucial molecules governing the phenotype transition of inactive Müller cells into activated states to acquire the above-mentioned phenotypes. Müller cell – T cell interactions were also inferred. The results show that activated Müller cells exhibit up-regulated expression of immune checkpoint molecules that primarily target Th1 cells, without displaying extra chemoattractive activities.

## Material and Methods

### Data Download

Single-cell sequencing (scRNA-seq) dataset was downloaded for re-analysis from Gene Expression Omnibus database (GSE132229). This dataset includes retinas from four Aire−/− mice and four wild type (WT) controls. The four female Aire−/− mice on a C57BL/6J background include two groups: two 11-week-old mice with grade 2 retinal inflammation and two 16-week-old mice with grade 3 retinal inflammation. The WT controls are age-matched and share the same genetic background. The grade 2 Aire−/− mice contribute 16,884 cells, the grade 3 Aire−/− mice contribute 12,640 cells, and the WT littermate controls contribute 34,672 cells (17).

### Single cell sequencing data process

The Seurat package (version 5.1.0) was used to perform data quality control and preprocessing following the standard protocol (25, 26). After quality controlling to remove outliers in features, counts and mitochondrial gene percentage, the sequencing data were integrated using the Harmony R package (version 1.2.3) (27). Data were normalized using the “LogNormalize” method in Seurat with a scaling factor of 10,000. Centering and scaling the data for 2,000 highly variable genes was performed using the “ScaleData” function.

### Dimension reduction, clustering and visualization

As described previously, these steps were performed using Seurat functions (28). Briefly, cells were clustered based on Principal Component Analysis (PCA) using the “RunPCA” function. The first 20 PCs identified (by Elbow method) were used in the “FindNeighbors” (based on k-nearest neighbor (KNN) graphs) and “FindCluster” (Louvain algorithm) functions. “RunTSNE” and “RunUMAP” functions were used with “pca” as the reduction method, to visualize the data. After clustering the retinal cells, Müller cell and T cells were re-clustered using the “subset” function, through combination of the “idents” and “gene expression” arguments. Rlbp1 >0 was used for Müller cells, while Cd3e > 0 was used for T cells. After performing “subset’, we re-ran the standard workflow shown above to cluster Müller cells along with T cells.

### Trajectory analysis and regulon network inference

For trajectory analysis of Müller cells, we utilized the Monocle3 package (version 1.3.7), a widely used computational tool to infer and visualize cell phenotype transition over time (29). Following trajectory analysis, SCENIC was used to identify transcription factors (TFs) governing the transition process, based on regulon co-expression patterns (30).

### Differentially Expressed Gene (DEG) analysis

To identify genes differentially expressed between cell clusters, we used the “FindMarkers” functions in Seurat and set log2FC >1 and p < 0.05 as criteria to define the significant changes in gene expression. Ingenuity Pathway Analysis (IPA) was used to predict the up-stream regulators driving gene expression changes. Gene Set Enrichment Analysis (GSEA) was performed using ClusterProfiler (31).

### Characterizing Müller cell subpopulations using SCassist

To gain insight into the features of each Müller cell subpopulation, we employed SCassist, a home-developed AI based tool. It takes the Seurat single cell object and related outputs, generates relevant data metrics, combines it with the template prompt, submits the augmented prompt to the LLM, parses the LLM’s response, displays the results or save them in a file or appends them to the Seurat object. Based on SCassist’s recommendation we selected ten pathways with the highest percentage of contributing cells to construct a comparison matrix. It contains module scores for each selected pathway in each Müller cell subgroup, quantifying their involvement within each subgroup. The module scores were calculated using a R package UCell (version 2.8.0) (32).

### Inferring interactions between Müller cells and T cells

To investigate the interactions between Müller cell subpopulations and T cell subpopulations, we utilized the R package CellChat (version 2.1.2), which calculates the potential interaction scores and the strength of cell–cell communications largely based on the expression levels of ligands and receptors (33). This analysis enabled us to identify the most significant ligand-receptor pairs between Müller cells and T cells, revealing the potential molecular mechanism underlying Müller cell-T cell interaction.

### Statistical analysis

All statistical analyses were performed using the algorithms associated with the R packages used, primarily the Wilcoxon Rank Sum Test for ‘FindMarkers’ in Seurat and UCell, Fisher’s exact test and hypergeometric test for ClusterProfiler, and Random Forest Regression and Benjamini-Hochberg test for SCENIC.

## Results

### Müller cells demonstrate a significant reproportioning process in the inflamed retina

**Figure 1.**
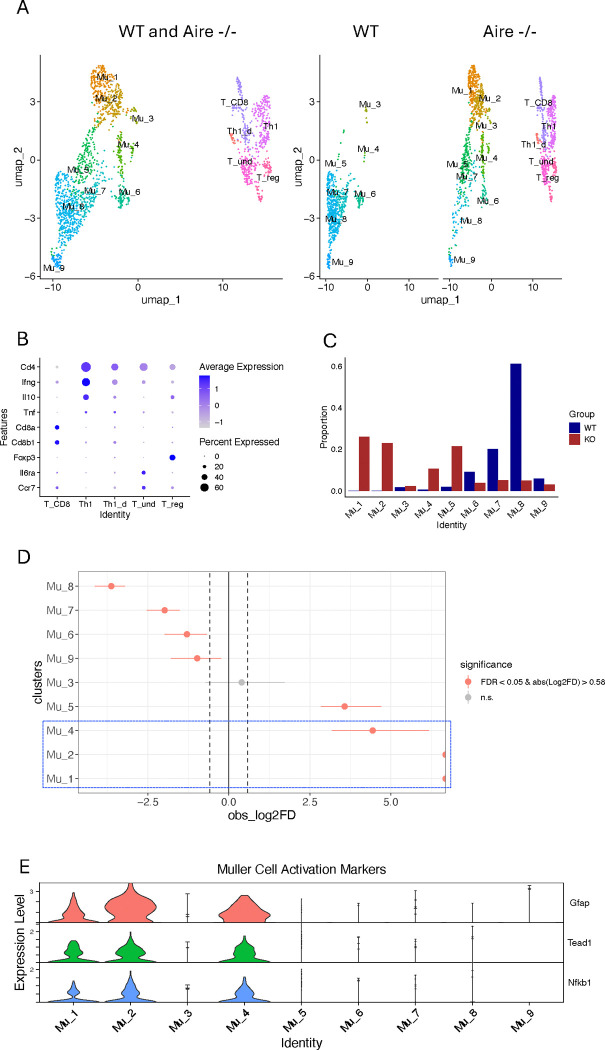
Re-clustering Müller and T cells from Aire−/− retina exhibits significant cell number changes in Müller cell subgroups compared to the control. (A) A UMAP plot showing nine subgroups of Müller cells and five subgroups of T cells from Aire−/− retinas, using WT retinas as the control. (B) Seurat dot plot showing expression levels of the marker genes used to group T cells. (C) Proportions of Müller cells subgroups from Aire−/− retinas and WT controls. (D) Proportion test showing statistical significance of cell number changes in eight subgroups of Müller cells compared to WT controls. (E) Expression of the marker genes in Aire−/− retina to distinguish activated Müller cells from the inactive Müller cells.

Re-clustering of the Müller and T cells from the inflamed retinas and naive controls identified nine distinct Müller cell subpopulations alongside five classic T cell subpopulations, comprising a total of 1,336 Müller cells and 568 T cells (Figure 1A). Using the reported gene markers for T cell subpopulations, four T cell subgroups were annotated: Th1, T_CD8, Treg and undifferentiated T cells (T_und) (17). A small group of CD4+ T cells lacking specific markers for any other T cell subpopulations, but characterized by moderate expression of Ifng, comparable expression of Tnf, and lower expression of Il10 compared to conventional Th1 cells, was annotated as Il10-deficient Th1 (Th1_d) (Fig1B). Following re-clustering, we examined Müller cell numbers across subgroups between Aire−/− retina and WT control to investigate the dynamic changes of the Müller cell populations during inflammation (Figure 1, C and D). As predicted, we noticed that the inflammation increases the diversity of Müller cells, leading to an expansion of the Müller cell subgroups from seven in WT to nine in Aire−/− retina, with the emergence of two new subgroups, Mu_1 and Mu_2 (Figure 1, A, C and D). The proportion test reveals a significant increase in cell numbers in Mu_1, Mu_2, Mu_4, and Mu_5 in the Aire−/− retina compared to WT, along with a significant decrease in Mu_9, Mu_6, Mu_7, and Mu_8, in that order (Figure 1, C and D). In contrast, Mu_3 does not exhibit significant changes in cell numbers during inflammation. A cross-condition comparison (Aire−/− vs WT) of gene expression levels of Mu_3 did not reveal any significant changes. In WT Müller cells, Mu_8 alone accounts for over 60% of the cell numbers, while in the Aire−/− retina, the top 3 subgroups with the most significant cell number increase (Mu_1, Mu_2, and Mu_4) comprise over 60% of the total Müller cell population. Notably, Mu_1, Mu_2 and Mu_4 are characterized by expression of Gfap, Tead1 and Nfkb1, which serve as reliable markers for mouse Müller cell activation (Figure 1E) (34). The log2FD values for Mu_1 and Mu_2 reach the maximum limit of the x-axis, indicating that these cell subpopulations are newly emerged, while Mu_4 also present in WT retina, consists of only a very small group of cells (Figure 1, A and D).

### SCassist reveals functional diversity among newly identified Müller cells in the inflamed retina

**Figure 2.**
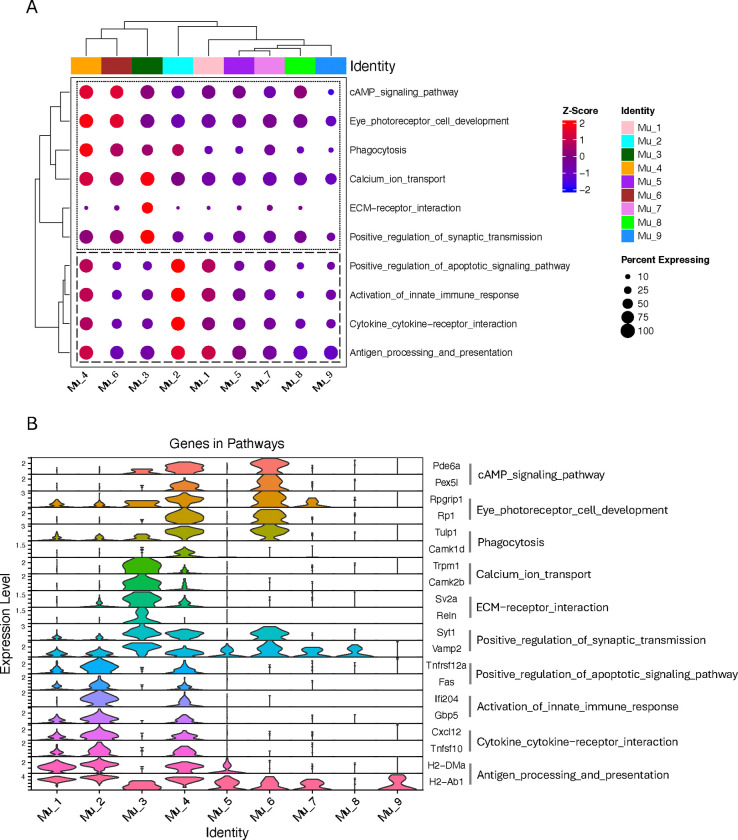
The comparison matrix used to quantify the involvement of pathways in each Müller cell subgroup from Aire−/− retina. (A) The comparison matrix includes module scores for each pathway across subgroups, calculated primarily based on the expression levels of genes associated with the pathways within each subgroup. Top section (function section) includes pathway for Müller cell functions. Bottom section (inflammation section) includes pathways relevant to inflammation. (B) The representative genes used to calculate the module scores for each pathway.

To characterize the features of each Müller cell subpopulation in the inflamed retina, Seurat identified over 40,000 overlapped marker genes across the nine subpopulations. After analyzing these genes, SCassist recommended 42 pathways from the hundreds of enriched pathways in KEGG or Gene Ontology highlighting the features of each subpopulation. We narrowed this list down to 10 pathways with the highest percentage of contributing cells to construct a comparison matrix for Aire−/− Müller cells. The comparison matrix contains module scores calculated primarily based on the expression levels of genes associated with the pathways in each subpopulation, thereby quantifying the involvement of the pathways within each subpopulation (Figure 2A). Notably, based on the information provided by SCassist, the pathways automatically group into two functionally distinct sections in the comparison matrix. The top section highlights the established functions of Müller cells, while the bottom section focuses on their responses to inflammation. Also as expected, the clustering patterns of the columns shown in the comparative matrix logically correspond to the spatial relationships observed in the UMAP (Figure 1A), despite the vastly different data scales involved (hundreds of genes provided by SCassist for comparison matrix versus thousands of genes used for the UMAP).

The activated Müller cell subpopulation, Mu_1, Mu_2 and Mu_4, exhibit higher module scores than others with Mu_2 being the most prominent for the pathways in the inflammation section, representing the most active response to inflammatory stimuli as reported previously (17–20) (Figure 2, A and B). Tnfsf10 and CC/CXC chemokines are the primary contributors to enrichment of the “cytokine-cytokine receptor interactions” pathway, while interferon-induced proteins play a key role in the enrichment of the “innate immune response.” MHC class II molecules and TAP complex components are essential for the enrichment of the “antigen processing and presentation” pathway (Figure 2B). Among the CC and CXC chemokines, we identified Ccl2, CCl27a, Ccl4, Ccl5, Ccl7, Ccl8, Cxcl10, Cxcl12 and Cxcl16 in the Aire−/− Müller cell subgroups but only CCl27a, Cxcl10 and Cxcl16 in the WT Müller cells (Figure 6C and Figure S1). Despite the similar trend in the inflammatory section for the activated Müller cells, they behave distinctly in the function section of the comparison matrix, where Mu_4 continues to exhibit high module scores indicating the augmented Müller cell functions, while module scores are lower for Mu_1 and Mu_2. The critical genes in this section, which contribute to the pathway enrichment, have been reported to be essential for Müller cells by encoding proteins maintaining retina homeostasis, such as Pde6a and Pex5l for cAMP signaling, Trpm1 and Camk2b for calcium transport, and Syt1 and Vamp2 for synaptic transmission (4, 8, 35) (Supplemental table). In addition, Mu_8 represents the most dynamic subpopulation, where the most dramatic cell number changes occur during inflammation (Figure 1, C and D). Mu_8 cells observed in this comparison matrix are the remnants of those inactive Müller cells (deficient of activation markers, figure 1E), which constitute the largest population of Müller cells before inflammation onset. Notably, Mu_3, Mu_4, Mu_5 represent the rare populations in WT retina with the similar pattern of module scores (Figure S1).

### Müller cells exhibit a profound phenotype transition in response to retinal inflammation

**Figure 3.**
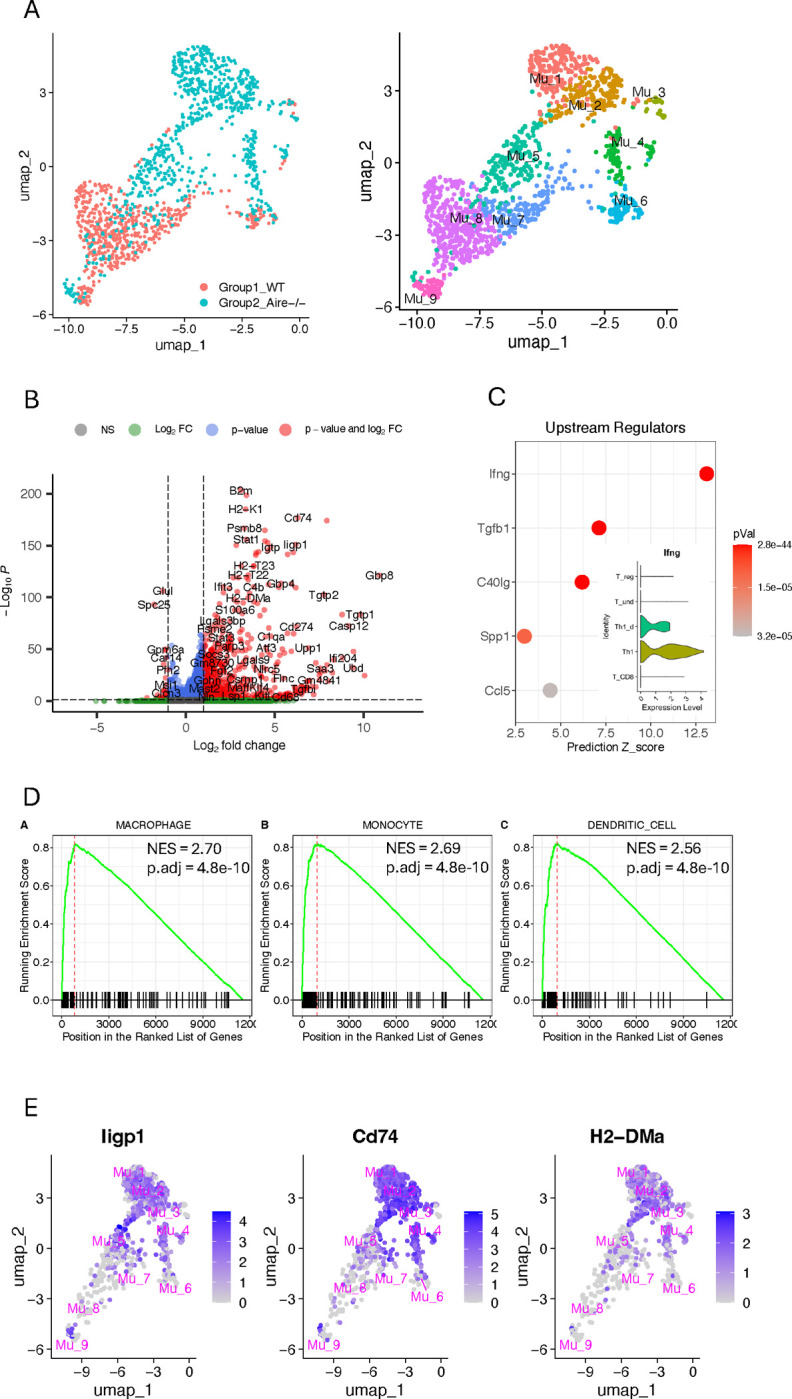
Cross-condition comparison of gene expression levels between Müller cells from Aire−/− retinas and WT controls. (A) Shift of distribution for Müller cells from Aire−/− retina compared to WT control in UMAP. (B) Significant asymmetrical gene expression changes in Aire−/− Müller cells compared to WT controls. (C) Predicted up-stream regulators using IPA, driving gene expression changes in Aire−/− Müller cells, and the sources of IFNg (inset). (D) GSEA analysis supporting the new phenotype of Müller cells in Aire−/− retinas globally. (E) Expression of the important genes significantly upregulated in Figure 3B, which are primarily identified in activated Müller cells.

The activated Müller cell subpopulation, Mu_1, Mu_2 and Mu_4, account for majority of the Müller cells in the inflamed retina (Figure 1C). In the comparison matrix, these cells exhibit significant involvement in the activities of antigen presentation, cytokine/chemokine interactions with their receptors, and innate immune response, which feature a macrophage-like phenotype (Figure 2A). To confirm this information obtained from comparison matrix, we leveraged the scRNA-seq data to perform a cross-condition comparison of gene expression levels between Müller cells in Aire−/− retina and those in WT control. It revealed that approximately 40% of genes in Aire−/− Müller cells exhibit significant asymmetrical expression changes compared to WT controls. Among them, 781 genes have over two folds upregulation and 30 genes have over two folds downregulation (Figure 3, A and B). Prediction analysis of upstream regulators driving these gene expression changes identified Ifng as the top driving force (Figure 3C). Notably, Th1, a major component of the T cell population in the inflamed retina of this mouse model, represents the primary source of IFNg, although Th1_d cells also contribute to IFNg production (Figure 1B and Figure 3C, inset panel). To identify potential new phenotypes of Aire−/− Müller cells resulting from these extensive and significant changes in gene expression, we conducted GSEA analysis using the cell-type-signature gene sets in MSigDB. The results indicate that Müller cells in the inflamed retina undergo a global phenotypic shift toward a macrophage or dendritic-cell-like phenotype, as demonstrated by their significant expression of marker genes associated with macrophages or dendritic cells (Figure 3D). The primary genes contributing to the new phenotype are confined in those activated Müller cells, indicating that the macrophage-like phenotype inferred from GSEA is primarily associated with activated Müller cells, which is in line with the observation from comparison matrix (Figure 2A and Figure 3, B and E).

### Trajectory analysis revealed two paths leading to distinct activated Müller cells in the inflamed retina

**Figure 4.**
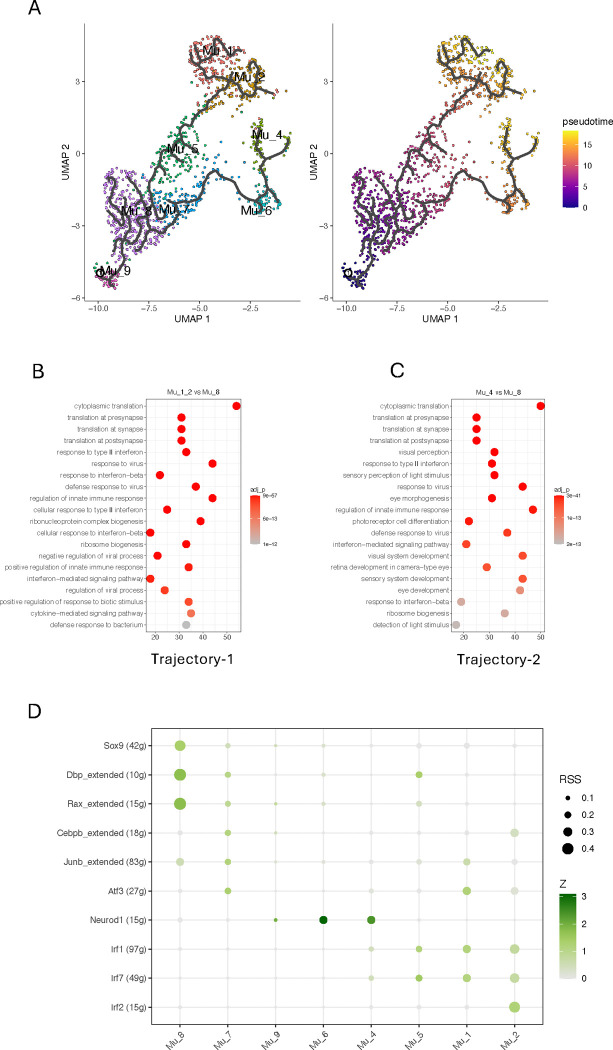
Trajectory analysis of Müller cell phenotypic transition in Aire−/− retina. (A) Pseudo-time trajectories of Müller cells in Aire−/− retina, leading to activated Müller cells. (B) Classes of top 20 genes with significant express changes in trajectory-1, from Mu_8 to Mu_1 and Mu_2. (C) Classes of top 20 genes with significant express changes in trajectory-2, from Mu_8 to Mu_4. (D) Top 10 regulons inferred by SCENIC, which drive the phenotypic transition of inactive Müller cells along the two trajectories into activated states.

To further explore the details of the phenotypic changes for Müller cells in the inflamed retina, we used Monocle3 to perform a trajectory analysis, tracking phenotypic transition through each population over time. It revealed that Mu_8 cells diverge into two primary branches: trajectory-1 progresses from Mu_8 to Mu_1 and Mu_2 via Mu_5, while trajectory-2 extends from Mu_8 to Mu_4 via M_6 and Mu_7 (Figure 4A). This is consistent with another observation from comparison matrix, where Mu_1 and Mu_2 display similar pattern of module scores, while Mu_6 is grouped with Mu_4 (Figure 2A). Mu_3 not only shows no difference in cell number compared to WT but also exhibits no significant changes in gene expression, suggesting that this group of cells may not undergo discernible phenotypic alterations (Figure 1, C and D). Along trajectory-1, Müller cells undergo significant changes in the expression of 606 genes, leading to an activated phenotype for Mu_1 and Mu_2, while, along trajectory-2, Müller cells exhibit significant changes in the expression of 872 genes, resulting in an activated Mu_4 phenotype (Figure S2). Classification of these genes using Gene Ontology (GO) reveals that the up-regulated genes in trajectory-1 are primarily pertinent to protein synthesis and inflammatory responses, while the up-regulated genes in trajectory-2 include additional genes involved in eye development and light perception, aligning with the information from comparison matrix revealing that Mu_1 and Mu_2 exhibit high scores for inflammation pathways, while Mu_4 show high cores for photoreceptor development as well (Figure 4B and Figure 2A).

We utilized SCENIC to identify the regulon networks governing the processes along these two trajectories with a focus on the top ten regulons (Figure 4D). In trajectory-1, Mu_5 exhibits increased activity of Irf1 and Irf7 compared to Mu_8 as the baseline. This is consistent with the observation in comparison matrix, where Mu_5 has begun to exhibit moderate scores in the inflammation section (Figure 2A). With the phenotype transition progressing, Mu_1 displays increased activities of Junb and Atf3, while Mu_2 displays increased activities of Irf2 and Cebpb, in addition to increased activity of Irf1 and Irf7 from the baseline. In trajectory-2, Mu_6 presents augmented Neurod1 activity compared to Mu_8 and Mu_7, which aligns with its performance in function section of the comparison matrix, such as the high activity in the pathway for photoreceptor development (Figure 2A). Following Mu_6, Irf1 and Irf7 take effect, completing the phenotype transition along this trajectory to Mu_4. Neurod1 distinguishes trajectory-2 from trajectory-1.

### Activated Müller cells demonstrate the most extensive interactions with T cells and other Müller cell subpopulations

**Figure 5.**
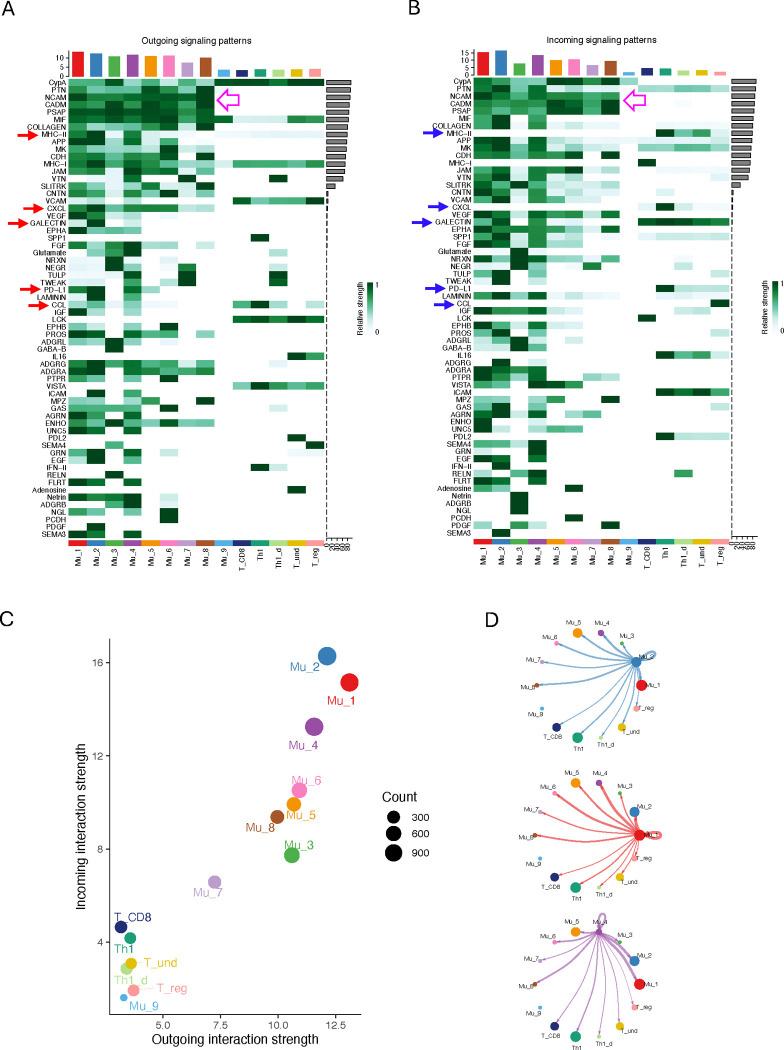
The inferred interactions among Müller cells and T cells in Aire−/− retina. (A) and (B) The sixty-two LR pairs were inferred among all Müller cells and T cells. Red arrows indicate the outgoing signals from the activated Müller cells with the receptor in T cells (blue arrows). Hollow pink arrows specify the interactions between Müller cells, which are mediated primarily by the common adhesive molecules. (C) A scatterplot showing the strength of outgoing and incoming signals for all the Müller and T cell subgroups. (D) The circle plots showing interactions between the activated Müller cells and other cell subgroups.

The reproportioned Müller cells, especially the newly emerged subgroups, characterized for their distinct features by SCassist, may exert novel and significant biological effects on the inflamed retina. To gain insight into these possibilities, we inferred the interactions between Müller cells and infiltrated T cells, as well as those within Müller or T cell subgroups, using a computational package CellChat. A total of 62 categories of ligand-receptor pairs (LR pairs) were identified including Cell-Cell Adhesion, Secreted Signaling and ECM-Receptors according to the annotation by CellChat (33) (Figure 5A). Key LR pairs in the Cell-Cell Adhesion category include NCAM, JAM, CADM, ICAM, MHCI/II, Galectin and PD-L1, while those in the Secreted Signaling category mainly encompass cytokines, chemokines and growth factors. In the scatter plot quantifying the strength of outgoing and incoming signals, the three activated Müller cell subpopulations, Mu_1, Mu_2 and Mu_4, exhibit the highest scores, with Mu_2 cells ranking highest, indicating that cells in these subgroups engage in the most extensive interactions with other cells beside themselves (Figure 5, C and D). Of the 62 categories of ligand-receptor (LR) pairs identified, 58 are present in the interactions between activated Müller cells and T cells, with LCK, IL6, PCDH and PDL-2 being absent for both incoming and outgoing signals. In contrast to the activated Müller cells, other Müller cell subgroups exhibit lower levels of interactions, with Mu_9 ranking the lowest. T cells, in contrast to majority of Müller cells, are notably less active in both sending and receiving signals. Within T cell subgroups, as expected, Th1 and CD8 T cells exhibit the highest activities, while Treg cells are the least active.

### Activated Müller cells dominate the checkpoint inhibition on Th1 cells

**Figure 6.**
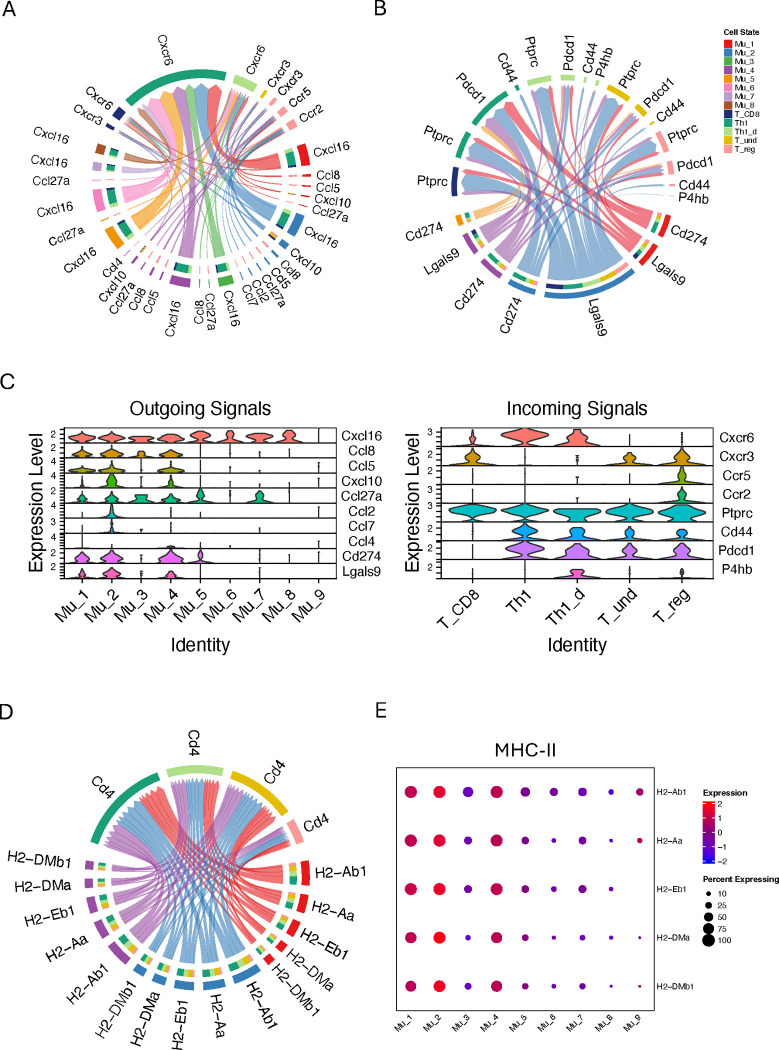
The inferred interactions between activated Müller cells and T cells through LR pairs of CCL, CXC, PD-L1, Galectin and MHC-II in Aire−/− retina. (A) Activated Müller cells interact with T cells through LR pairs of CCL and CXC. (B) Activated Müller cells interact with T cells through LR pairs of PD-L1 and Galectin. (C) Seurat violin plots showing expression levels of the genes mediating outgoing signals from Müller cells or incoming signals to T cells. (D) Activated Müller cells interact with T cells through LR pairs of MHC-II. (E) A dot plot showing the expression levels of MHC-II molecules in Müller cell subgroups.

We are particularly interested in how Müller cells influence the prognosis of retinal inflammation; therefore, we focused primarily on the interactions between activated Müller cells and T cells. Five categories of interactions, including CCL, CXCL, MHC-II, GALECTIN and PD-L1, were identified to have both activated-Müller-cell specific outgoing signals (Figure 5A, red arrows) and corresponding receptor signals in T cells (Figure 5B, blue arrows). Mu_1, Mu_2 and Mu_4 commonly produce Cxcl16, Cxcl10, Ccl5, Ccl8, and Ccl27, with Cxcl16 exhibiting the most potent signals due to high expression of Cxcl16 from Müller cells and Cxcr6 from Th1 cells (Figure 6, A and C). Among them, Cxcl10, Ccl5 and Ccl8 are relatively specific to activated Müller cells, while Cxcl16 and Ccl27 can be produced from other Müller cells at even slightly higher levels for Cxcl16. In addition to these three activated-Müller-cell specific chemokines, Mu_2 cells can also produce Ccl2 and Ccl7, while Mu_4 cells can make Ccl4, at marginal levels. On the recipient side, Th1 and Th1_d cells are exclusively targeted, through Cxcr6, by Cxcl16, whereas other T cell can respond to multiple chemokines. Notably, activated Müller cells do not exhibit extra chemoattraction to Th1 cells compared to other Müller cells deficient of activation markers (Figure 6A). The activated Müller cells not only produce chemokines but also express checkpoint inhibitors to suppress the T cells (Figure 6B). Specifically, these checkpoint inhibitors include Cd274 and Lgals9. Importantly, these checkpoint inhibitors are predominantly expressed in the activated Müller cells, and Th1 cells are their predominant targets (Figure 6, B and C). Lgals9 expressed by Mu_2 cells exerts the most potent inhibitory signals to the T cells. The analysis also reveals that the activated Müller cells express higher levels of MHC II molecules than other Müller cell subgroups, specifically H2-Ab1, H2-Aa, H2-Eb1, H2-DMa, and H2-DMb1, to interact with CD4+ cells (Fig. 6D and E).

## Discussion

In this article, we provide the first scRNA-seq analysis of Müller cells in the retina with autoimmune inflammation, characterizing the distinct features of Müller cell subgroups in response to inflammatory stimuli. SCassist, our in-house developed AI based tool, played a key role in functional analysis identifying characteristic pathways and associated genes reported in this study. Our major findings include 1) retinal inflammation triggers dramatic reproportion of Müller cells and the phenotype transition, 2) nine subgroups of Müller cells are identified, three of which are activated Müller cells characterized by macrophage-like properties with or without augmented Müller cell functions, 3) Müller cells follow two trajectories to acquire activated Müller cell phenotypes, which diverge due to differences in activities of Neurod1 and Irf family TFs, 4) activated Müller cells do not show extra chemoattraction to Th1 cells but exhibit nearly exclusive expression of immune checkpoint molecules primarily targeting Th1 cells.

While mouse Müller cells are generally regarded as relatively homogeneous compared to those in the chicken retina, their homogeneity does change in response to retinal injuries (34, 36, 37). Two-subpopulation model of Müller cells have been proposed in previous studies on the damaged mouse retina; however, this model could be overly simplistic, given the recently emerging large body of scRNA-seq data on zebrafish retina highlighting the complexity of Müller cell composition (34, 38). Particularly in mouse retina with autoimmune inflammation, little is known regarding the subgroups of Müller cells. In this work, after subsetting and re-clustering Müller and T cells from Aire−/− retina, we identified nine distinct Müller cell subpopulations along with five traditionally classified T cell subgroups. When comparing the cell numbers in Müller cell subpopulations between Aire−/− and age-matched WT controls, we observed significant proportional changes across all subgroups except Mu_3 (Figure 1, C and D). This comparison identified Mu_1 and Mu_2, two distinct activated Müller cell subgroups, as newly emerged cell populations resulting from retinal inflammation. The activated Müller cells, those in Mu_1, Mu_2, and Mu_4, comprise over 60% of the total Müller cells, likely accounting for the major effects on the retina caused by Müller cells. Therefore, these cells are the main focus in this analysis.

To characterize the functions of each Müller cell subpopulation identified in the inflamed retina, from 42 pathways recommended by SCassist, we selected 10 pathways to construct a comparison matrix, which clearly delineated the features of each Müller cell subgroup in a quantitative manner. The activated Müller cells present higher module scores than others in the inflammation section of comparison matrix, indicating the most active response to inflammatory stimuli (17–19, 21) (Figure 2A). The involved pathways, including “Antigen processing and presentation”, “Activation of innate immune response” as well as “Cytokine cytokine-receptor interactions”, offer a plausible explanation for why Müller cells in the Aire−/− retina exhibit a global macrophage or dendritic cell-like phenotype (Figure 3). IFNg was identified as the major up-stream signal driving the Müller cell phenotypic changes (Figure 3C). This is consistent with the nature of Air-deficiency-induced inflammation, which is a Th1 driven disease (17). Th1 cells are the well-known culprits for autoimmune retinal inflammation, as evidenced in other animal models as well (15, 39, 40). In addition to inflammatory response, Mu_4 cells, about 10% of total Müller cells in the inflamed retina, exhibit augmented Müller cell functions. However, it is not clear yet whether this is detrimental or beneficial for retinal homeostasis. In Zebra fish, upon retinal injury, Müller cells can de-differentiate into a neuron progenitor state to regenerate a new batch of retinal neurons for restoring retinal functions (34, 38). Although mouse Müller cell lost this capacity with evolution, such a potential still remains (34). This may partially explain why Mu_4 exhibits a high level of involvement in function section of the comparison matrix. Interestingly, WT Müller cells encompass a tiny group of Mu_4 cells, but it significantly expands during inflammation (Figure 1A and Figure S1). The pathophysiologic consequences of this change may warrant further investigation.

To further explore the information provided by SCassist, we investigate the phenotype transition paths using Monocle3, which is widely used to infer and visualize cell phenotype transition (29). Analysis reveals two trajectories diverging at Mu_8, ultimately giving rise to three groups of activated Müller cells (Figure 4A). Mu_8 is the largest subgroup of Müller cells before inflammation onset, where the cell number decreases most significantly during inflammation, suggesting that the activated Müller cells primarily begin from this point to undergo their phenotypic transition journey (Figure 1C). Trajectory-1 passing through Mu_5, leads to Mu_1 and Mu_2 (Figure 4A). Mu_5 exhibits the signs (moderate module scores) of early involvement of inflammatory pathways in the comparison matrix, which is primarily governed by Irf1 and Irf7, two TFs in Irf family responding to IFNg stimulation (Figure 4D). Differences in the TF activities of Irf family, Junb and Atf3 in Mu_1 and Mu_2 drive them into two distinct directions. High activity of Irf2, in addition to Irf1 and Irf7, in Mu_2 separates it from Mu_1 and explains why this group of cells are the most inflammatory cells, as revealed by comparison matrix (Figure 2A). Trajectory-2 distinguishes itself from trajectory-1 by high activity of Neurod1 in Mu_4 and Mu_6, a key factor for retinal neuronal differentiation (41) (Figure 2A). Irf1 and Irf7 further drive Mu_6 to Mu_4 transition. The neuronal properties of Mu_4 and Mu_6 are consistently supported by enriched Gene Ontology (GO) terms in trajectory analysis and high scores in function section of comparison matrix (Figure 4C and Figure 2A).

As shown in Figure 2A, the activated Müller cell subgroups exhibit distinct functions that differ from one another and from inactivated cells. As a result, these cells may play a significant role in influencing retinal physiology during retinal inflammation. Indeed, activated Müller cells display the most extensive interactions with other cells in the inflamed retina (Figure 5C). Here we particularly focused on the interactions between activated Müller cells and T cells, given their potential roles, suggested by the *in vitro* studies, in determining the prognosis of retinal inflammation (19, 22). Five categories of interactions were identified exerting interactions between active Müller cells and T cells, including CCL, CXCL, MHC-II, GALECTIN and PD-L1 (Figure 5, A and B). Interestingly, all detected CC chemokines, including Ccl2, Ccl4, Ccl5, Ccl7, and Ccl8, except Ccl27a, are primarily derived from activated Müller cells in Aire −/− retina, while CXC chemokines, Cxcl16 and Cxcl10, are present in both Aire −/− and WT retina. Particularly, Cxcl16 is present in all the Müller cell subgroups, including those in WT retina, except Mu_9, suggesting that Cxcl16 may play a crucial role in maintaining retinal homeostasis (42) (Figure 6C and Figure S1). We observed that activated Müller cells interact with Th1 cells exclusively through Cxcl16 and express this chemokine at a level similar to, if not lower than, that from other Müller cells (Figure 6, A and C; Figure S1). Therefore, activated Müller cells do not exhibit a greater capacity to chemoattract Th1 cells compared to other Müller cells. In contrast, checkpoint molecules are expressed almost exclusively in activated Müller cells (Figure 6C). Th1 cells have the greatest extent of being affected by these checkpoint molecules, making the activated Müller cells important in reducing Th1 cells activity. Additionally, we observed that activated Müller cells exhibit higher expression levels of MHC II molecules than other Müller cells (Figure 6, D and E). Despite previous studies on Müller cells at the bulk cellular level suggesting that overexpression of MHC-II could further activate T cells within the inflamed retina, several questions exist with this postulation (17, 18). First, previous *in vitro* studies demonstrated that T cell activation by antigen presentation from Müller cells was lost when inhibitory capacity of Müller cells was intact, which was determined by the integrity of a membrane bound molecule on Müller cells (19, 20). Second, it is not clear whether this membrane bound molecule is present or not on the activated Müller cells during retinal inflammation. Moreover, over presentation of antigen to T cells may not always be activating. There is recent evidence supporting that MHC-II molecules expressed by tumor cells may lead to T cell exhaustion (43). Taken together, this analysis sheds light on the novel role of activated Müller cells, suggesting that these cells may potentially restrain Th1 cell activities.

In summary, our study demonstrates the value of our AI system in enhancing scRNA-seq analyses in the study on autoimmune retinal inflammation by providing accurate information on the involved pathways and associated genes. The construction of a comparison matrix based on the AI provided information proved to be an efficient method for elucidating the features of cell subgroups, offering deeper insights into their biological significance and directing in-depth data analysis. With these tools at hand, we were able to extract crucial information from subgroups of Müller cells in inflamed retina, illustrating the dynamic process by which they transition into distinct activated Müller cells, exhibiting novel functions that were previously unrecognized. The discovery of Müller cells’ dynamic transformation into activated subpopulations provides new insights into the molecular mechanisms underlying autoimmune retinal inflammation, which may inform the development of novel therapeutic strategies for treating this debilitating disease.

## Supplementary Material

Supplement 1

Supplement 2
