# Supplementary material for: AI-Driven Analysis Unveils Functional Dynamics of Müller Cells in Retinal Autoimmune Inflammation
Source: bioRxiv. 2025 May 12:2025.02.28.640907. Preprint. [Version 3] doi: 10.1101/2025.02.28.640907 (PMC11908203; doi:10.1101/2025.02.28.640907)
Supplement: 1 [file NIHPP2025.02.28.640907V3-supplement-1.pdf]

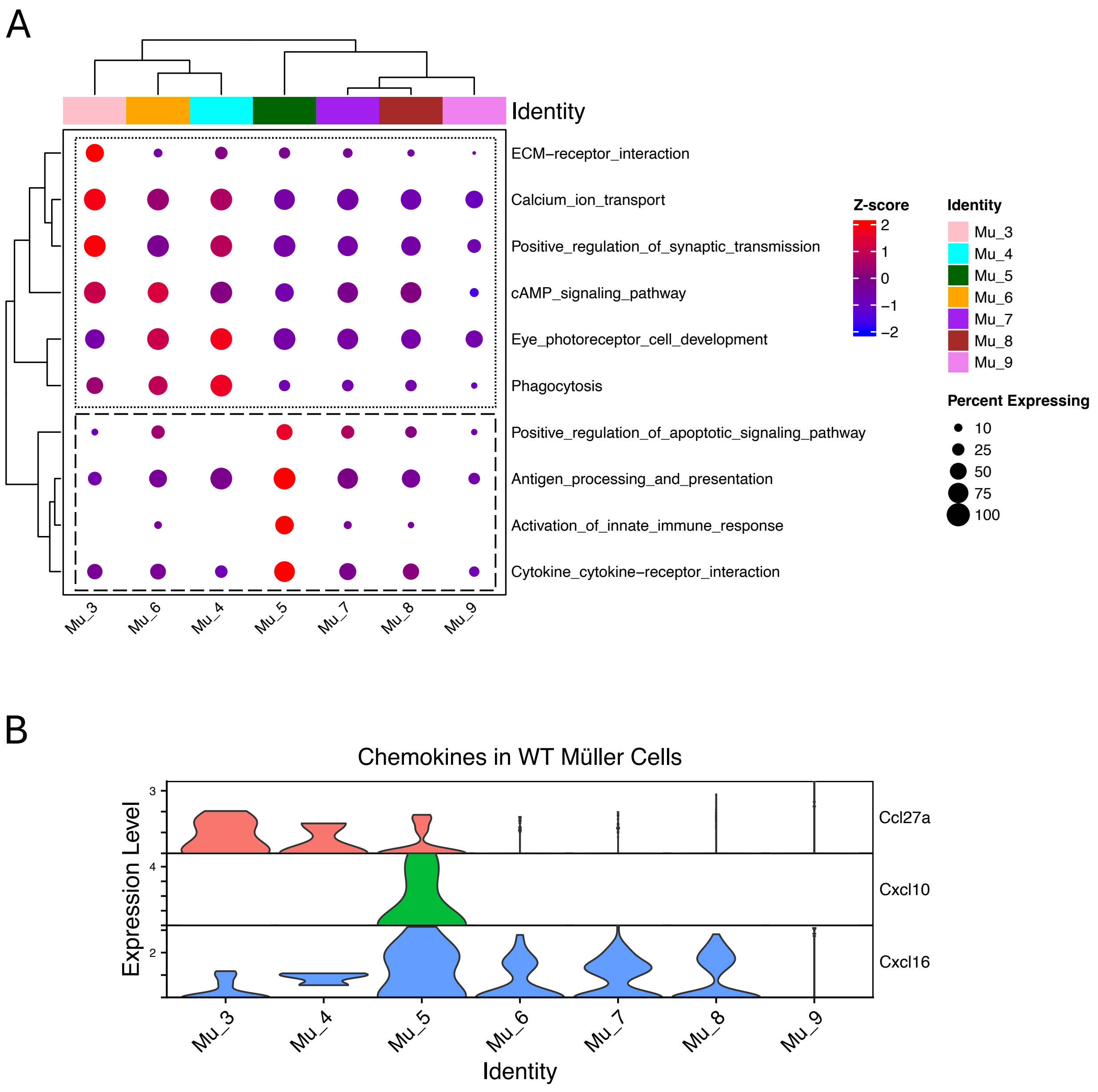

Figure S1. (A) Comparison matrix for the pathways in WT Müller cell subgroups. (B) Chemokine expression in WT Müller cell subgroups.

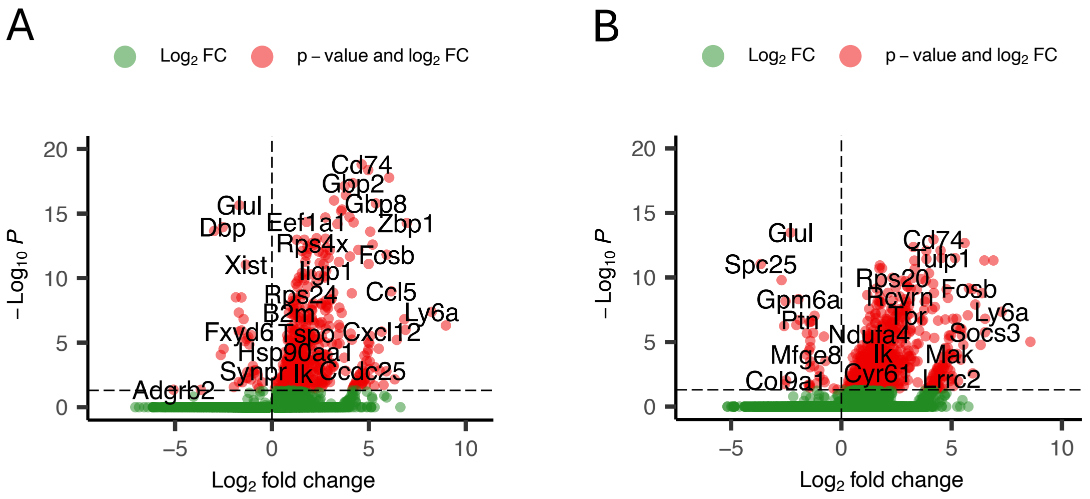

Figure S2. (A) Gene express changes in Trajectory-1. DEGs are identified in Mu_1 and Mu_2 (vs Mu_8). (B) Gene express changes in Trajectory-2. DEGs are identified in Mu_4 (vs Mu_8).
